# Focal cortical dysplasia type II: review of neuropathological manifestations and pathogenetic mechanisms

**DOI:** 10.1186/s42494-024-00195-y

**Published:** 2025-02-17

**Authors:** Yubao Fang, Yaqian Zhang, Tiancai Huang, Shengyu Yang, Yinchao Li, Liemin Zhou

**Affiliations:** Department of Neurology, The Seventh Affiliated Hospital of Sun Yat-sen University, Shenzhen, 518107 China

**Keywords:** Focal cortical dysplasia, Abnormal differentiation, Gene mutations, Epigenetic alterations

## Abstract

Focal cortical dysplasia (FCD) is an important cause of intractable epilepsy, with FCD type II (FCD II) being the most common subtype. FCD II is characterized by cortical dyslamination accompanied by dysmorphic neurons (DNs). Identifying the molecular alterations and targetable biomarkers is pivotal for developing therapies. Here, we provide a detailed description of the neuropathological manifestations of FCD II, including morphological alterations and immunophenotypic profiles, indicating that abnormal cells exhibit a diverse spectrum of mixed differentiation states. Furthermore, we summarize current research on the pathogenetic mechanisms, indicating that gene mutations, epigenetic alterations, cortical developmental protein disturbances, inflammatory processes, and extrinsic damages may lead to abnormal neuronal proliferation and migration, thereby contributing to the emergence and progression of FCD II. These findings not only enhance our understanding of the pathogenesis of FCD II but also offer new directions for clinical diagnosis and treatment. Future research should further explore the interactions among these factors and employ multidisciplinary approaches to advance our understanding of FCD II.

## Background

Focal cortical dysplasia (FCD), a subtype of malformations of cortical development (MCD) [1], is characterized by congenital structural and cytoarchitectural abnormalities of the six layers of the cerebral cortex [2]. It is a predominant cause of intractable epilepsy in the pediatric population [3], and the third leading etiology of refractory epilepsy in adults [4]. According to the latest International League Against Epilepsy (ILAE) classification, FCD is divided into five subtypes, with FCD II being the most common, accounting for 45.3% [5, 6]. FCD II is characterized by cortical dyslamination, dysmorphic neurons (DNs), reduced myelination in the white matter, and indistinct gray matter to white matter boundaries [7, 8]. And FCD IIb is distinguished from FCD IIa by the additional presence of balloon cells (BCs) and disrupted clusters of oligodendroglial cells [9].

Extensive research has been conducted on FCD II, including its histomorphology and pathology, neuroimaging, gene mutations, transcriptomics, proteomics, changes in metabolomics expression, EEG alterations, and abnormal cellular electrophysiological characteristics [10–15]. However, FCD II still presents challenges in diagnosis and treatment.

In this review, we summarize the neuropathological features of FCD II, including morphological alterations and immunophenotypic profiles. Furthermore, we explore the pathogenetic mechanisms associated with FCD II, including gene mutations, epigenetic alterations, cortical developmental protein disturbances, inflammatory processes, and extrinsic damages. Summarizing these neuropathological manifestations and pathogenetic mechanisms can help clinical diagnosis and provide direction for future treatment.

## Clinical challenges of FCD II

As the most common type of cortical dysplasia in epilepsy surgery, FCD II is typically with an average onset age of 5 years for seizures. Among confirmed cases, 51% are localized to the frontal lobe [5]. Compared to patients with FCD IIa, those with FCD IIb exhibit a shorter duration of symptoms, higher seizure frequency, and younger mean age at surgery, indicating greater severity of the seizure syndrome [16]. Combining radiological and histopathological findings with genetic information has introduced a new classification for FCD II called bottom-of-sulcus dysplasia (BOSD), which refers to lesions anatomically confined to the bottom of the sulcus [6].

It has been indicated that both ictal discharges and interictal rhythmic spiking originate from the dysplastic areas characterized by DNs [17, 18]. In addition, researches have shown that phase-amplitude coupling, spikes, fast gamma and ripples are related to the density of DNs and help localize the seizure onset zone [19–21]. Possible epileptogenic mechanisms include abnormal N-methyl-D-aspartate (NMDA) receptors, alteration in GABAergic neurons and abnormality of NKCC1/KCC2, pacemaker GABA synaptic activity, and the reorganization of the perisomatic inhibitory system [22–26]. In addition, aberrant adenosine signaling, endoplasmic reticulum stress, unfolded protein accumulation, and neuroinflammation can also contribute to the imbalance in the central nervous system’s excitation-inhibition system [27–29].

While the complex relationship between areas of developmental abnormality and the occurrence of epileptic seizures is progressively being elucidated, and resection epileptogenic tissue is essential for significantly improving seizure outcomes, determining clear margins remains a challenge [30]. Firstly, despite some FCD II cases showing significant changes on MRI, such as the transmantle sign and black line sign, MRI remains negative in 1/3 to 1/2 of FCD II patients, leading to unsatisfactory postoperative seizure-free rates and a poor prognosis [31–36]. Secondly, the region exhibiting the greatest cortical dysplasia does not necessarily align with the area most prone to epileptic activity [37]. Dysplastic tissue can also extend microscopically beyond the visible lesion or alter the epileptogenic threshold of adjacent cortical tissue [38, 39]. In addition to surgery, drugs for the treatment of FCD II also in constant developments. MTOR (mechanistic target of rapamycin) inhibitors have emerged as a novel therapeutic option for targeting FCD-related seizures, as 60% of FCD II cases, particularly FCD IIb, display gene mutations in the mTOR signaling pathway [40]. However, the efficacy of treatment with the mTOR inhibitor remains controversial [41, 42].

Therefore, identifying the molecular alterations and targetable biomarkers of DNs and BCs is crucial to understanding the pathogenesis of FCD II and developing targeted therapies [43].

## Neuropathological manifestations of FCD II

### Morphological alterations in FCD II

**Fig. 1:**
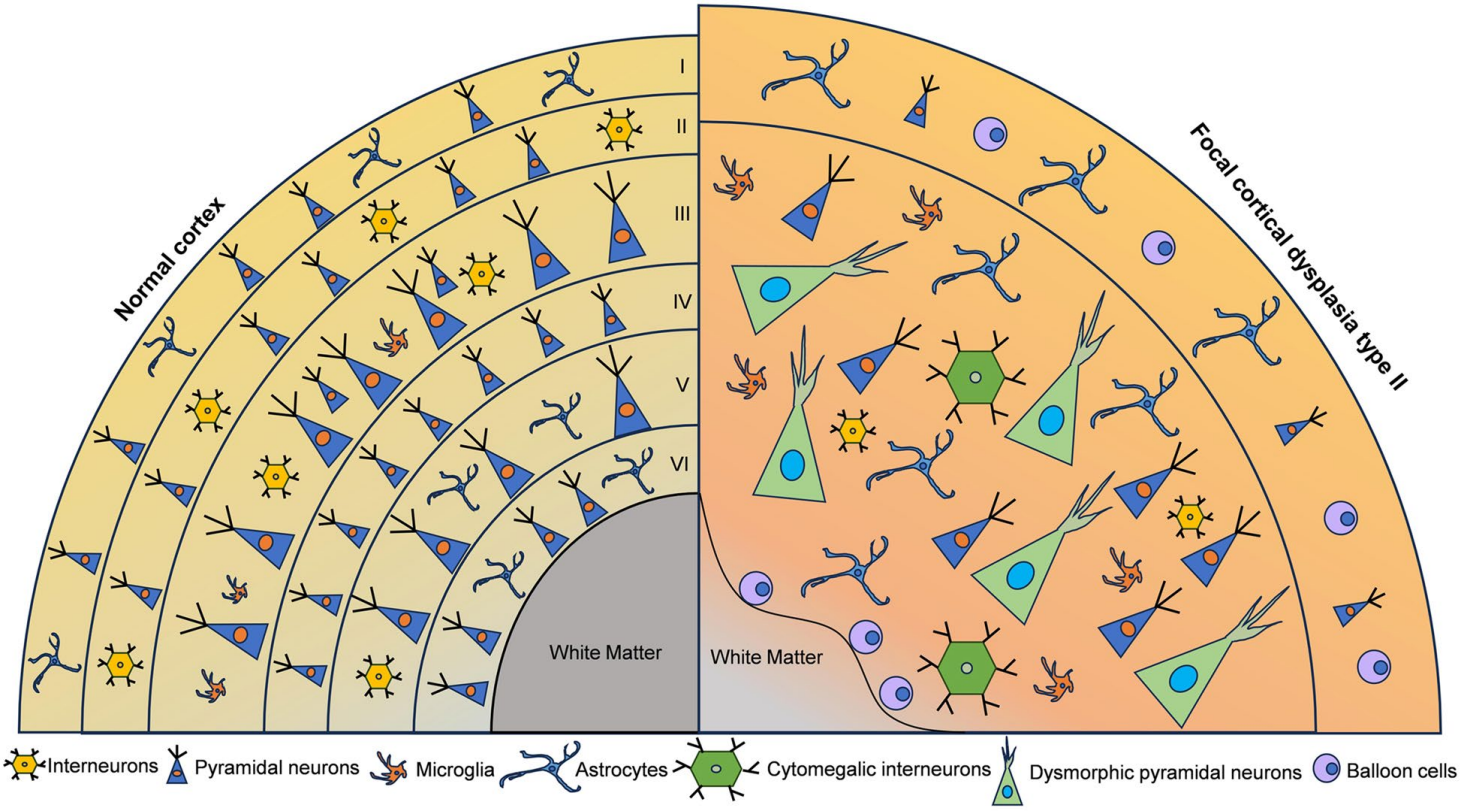
The schematic depicts the architectural and cellular abnormalities in FCD II. In FCD II, lamination is virtually absent, except for an expanded layer I. Dysmorphic pyramidal neurons and cytomegalic interneurons are dispersed throughout layers II to VI, frequently extending into the white matter. In FCD IIb, BCs are predominantly observed in the upper layers and within the white matter

In FCD II, the six-layered homotypic structure typically disintegrates when mixed with normal pyramidal cells, while cortical layer I often remains well-defined but broadened [44]. And the impacted neocortex also experiences a decrease in cell density, which is more pronounced in FCD IIb compared to FCD IIa [9]. However, concurrent research indicates the presence of hidden cortical lamination involving normal-looking neurons, which retain their ability to migrate correctly within the cortex, whereas DNs exhibit alterations in their migratory patterns [45]. In addition to observing a reduction in myelin staining in the underlying white matter, studies at the ultrastructural level have found that the myelin sheaths of layer V axons are thinner in dysplastic specimens compared to controls (Fig. 1). This indicates that myelination is also compromised in the gray matter of the dysplastic area [46]. Furthermore, DNs and BCs predominantly accumulate at the base of the sulcus, with their numbers gradually diminishing along the ascending cortex [47].

DNs, first described by Crome [48] and Taylor [49], can be disseminated across the full extent of the cortical architecture or embedded within the white matter [7]. They are distinctly delineated by a constellation of marked cytological aberrations: both the neuronal cell diameters and the cell nucleus diameter are significantly enlarged (the shortest diameter exceeds 25 μm, which is significantly larger than that of typical pyramidal cell in age- and location-matched postmortem controls [50]). Beyond the augmented somatic volume, DNs manifest morphological deviations characterized by abnormal thickness of the initial portion of apical dendrite and axon [51]. Despite morphological similarities in cell shape among distinct DNs, they may encompass a diversity of cellular phenotypes, as DNs primarily represent altered pyramidal neurons, but some may also exhibit features consistent with interneurons [52]. Utilizing a series of morphological parameters for the morphological reconstruction of brain tissue excised from FCD II patients, it suggests that DNs located primarily in the upper layers (layers II–IV) of the grey matter might contribute to the seizure genesis [53].

BCs exhibit a substantial cellular body with peripheral nuclei and enlarged, glassy cytoplasm, resembling the giant cells observed in tuberous sclerosis complex (TSC) [54]. These cells can be found in any cortical location, including layer I, and are frequently detected in the underlying white matter [55]. However, they always circumvent areas where dysmorphic neurons accumulate [9], and it has been confirmed that they are not the source of abnormal electrical activity [56].

### Immunophenotypic profile of FCD II

The immunophenotypic profiles of DNs and BCs have been extensively investigated, predominantly to elucidate their lineage and maturation processes [57].

Nissl substance is aggregated and displaced towards the cell membrane, while phosphorylated (antibody 2F11) and non-phosphorylated neurofilament isoforms (SMI-32) accumulate in the cytoplasm of DNs [7]. In contrast, BCs exhibit an opalescent, glassy, eosinophilic cytoplasm devoid of Nissl substance. Furthermore, BCs commonly accumulate intermediate filaments such as vimentin and nestin [58]. These markers signify the presence of cytoskeletal abnormalities within DNs and BCs [57].

DNs and BCs have been demonstrated to express certain stem cell markers. Elevated levels of nuclear receptor subfamily 2 group F member 1 (NR2F1) are observed in DNs, while both NR2F1 and sex-determining region Y-box 2 (SOX2) are expressed in BCs [59]. Furthermore, BCs also express a range of stem cell markers such as GFAP-delta [60], CD133 [61], beta-1 integrins [62], and the onco-fetal antigen CD34 [63]. Both BCs and pyramidal neurons in DNs express Otx1, phospho-vimentin, Pax6, and BLBP, but lack Dlx1/Dlx2 expression, suggesting that they are derived from radial glial cells (RGCs) in the telencephalic ventricular zone (VZ) [64]. Additionally, interneurons in DNs demonstrate calbindin and parvalbumin expression but lack calretinin, suggesting an origin from the medial ganglionic eminence [52].

The high expression of NKCC1 in DNs and the altered subcellular distribution of KCC2 support the hypothesis of impaired developmental maturation in FCD II [65]. Within the normal cerebral cortex, the progression of neuronal differentiation is characterized by the successive expression of proteins, starting with MAP1B, then followed by NF-H, culminating in the expression of NeuN. MAP1B, an immature neuronal marker, frequently co-localized with GFAP (a glial marker), whereas the mature neuronal marker NeuN rarely did [66–68]. In DNs and BCs, co-expression of MAP1B and GFAP can be observed. However, NeuN and GFAP are rarely co-localized in DNs, and only slightly more frequently in BCs [68]. Studies have found that immunoreactivity with NF-H is not observed in any BCs [69]. Additionally, BCs also express β-tubulin III, which is the earliest marker of postmitotic neurons [61].

In summary, the mixed glio-neuronal features imply that DNs and BCs exhibit a diverse spectrum of mixed differentiation states [68].

## The pathogenetic mechanisms of FCD II

Cellular proliferation within the mammalian cortex predominantly takes place in the germinal regions known as the VZ and subventricular zone (SVZ) [70]. In the initial stages, neuroepithelial progenitor cells within the VZ engage in mitotic activity to sustain the progenitor pool while concurrently giving rise to radial glial cells (RGCs), which are not only proliferative but also serve as migratory guides for neurons [71]. Following the formation of the first neuronal layer (the primordial plexiform layer or pre-plate), the SVZ forms between the VZ and pre-plate [72]. RGCs facilitate neurogenesis through successive self-renewal and asymmetric division, and that new-born neurons often use the parent cell’s radial fibre to migrate to the cortical plate [45, 73].

The immunophenotypic profile of BCs and pyramidal neurons in DNs indicates their derivation from RGCs in the telencephalic VZ [64].Together with immunophenotypic profile, the intrinsic membrane properties and synaptic features of DNs and BCs reveal developmental arrest and immaturity at both cellular and synaptic levels [74]. The co-expression of glial and neuronal antigens in different combinations indicates that the generation of aberrant cells in FCD II is associated with aberrant neuronal-glial differentiation [68, 75]. Concurrent studies have also suggested DNs exhibit alterations in their migratory patterns [45]. Collectively, these data suggest that the appearance of aberrant cells may be related to disruptions in the processes of neuronal-glial proliferation and migration, potentially occurring during the first half of gestation [45, 68]. The somatic mutation hypothesis posits that a somatic gene mutation occurs in a single progenitor cell during a critical phase of cortical development. This mutant cell continues to proliferate, producing progeny that carry the mutation. Consequently, DNs and BCs exhibit distinct morphological characteristics and are likely derived from different differentiations pathways of the same mutant neuroglial progenitor cell [76, 77]. This hypothesis would be consistent with the characteristic funnel-shaped morphological distribution of FCD II lesions, since clonally related neurons show the same distribution pattern [68, 78]. Furthermore, abnormalities in cellular migration may be the primary cause of pronounced MRI anomalies predominantly observed at the bottom of the sulcus. The tendency for BOSD to localize in the frontal sulci may be attributed to the shorter neurogenesis time required in rostral cortices compared to caudal areas [47].

**Fig. 2:**
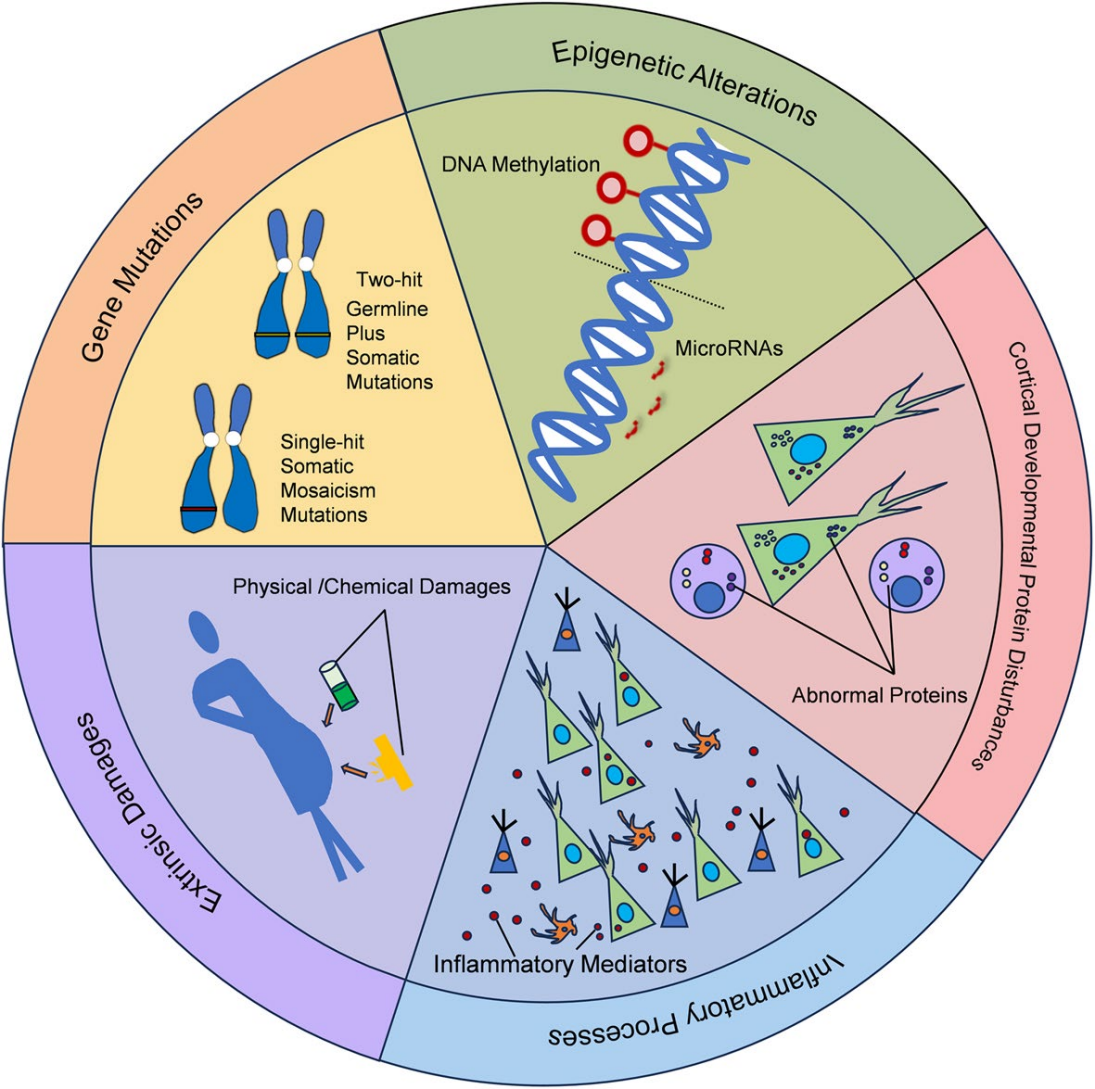
The pathogenic mechanisms in FCD II. Gene mutations, epigenetic alterations, cortical developmental protein disturbances, inflammatory processes, and extrinsic damages may contribute to the emergence and progression of FCD II

However, the X-androgen receptor (XAR) inactivation assay has detected varying lengths of XAR-CAG repeats in individual DNs and BCs. This suggests that DNs and BCs may originate from a mixed cell population derived from multiple progenitor cells [79]. Simultaneously, the pyramidal neurons and interneurons in DNs exhibit distinct origins [52]. These findings suggest DNs and BCs in FCD lesions are heterogeneous, likely arising from multiple progenitor cells or post-mitotic neurons that were extrinsically damaged during cortical development [68, 79]. Besides, some studies have suggested that the etiology of FCD may be partially attributed to the abnormal retention of pre-plate cells in the subplate and marginal zones. Cytomegalic neurons could be postnatally retained subplate cells. This phenomenon likely occurs during the advanced stages of cortical development, as it does not align with the expected pattern if severe cortical dysplasia were the result of developmental processes significantly altering early to mid-corticoneurogenesis [74, 80]. Thus, the pathological mechanisms underlying FCD II remain controversial (Fig. 2).

### Gene mutations

MTOR serves as an intricately conserved signaling nexus, integrating neuronal activity and a variety of synaptic inputs. MTOR is found in two functionally distinct complexes: mTORC1 and mTORC2, which are pivotal in regulating long-term synaptic efficacy and memory consolidation [81]. In addition, erturbations in mTOR signaling pathways are correlated with an array of neurodevelopmental and neuropsychiatric pathologies [82]. The cell count analysis confirmed that  phospho-S6 (pS6), a downstream target of the mTOR pathway, has been detected expressing in the majority of DNs, which indicates that the activation of the mTOR pathway plays a critical role in the formation of FCD II [45].

**Fig. 3:**
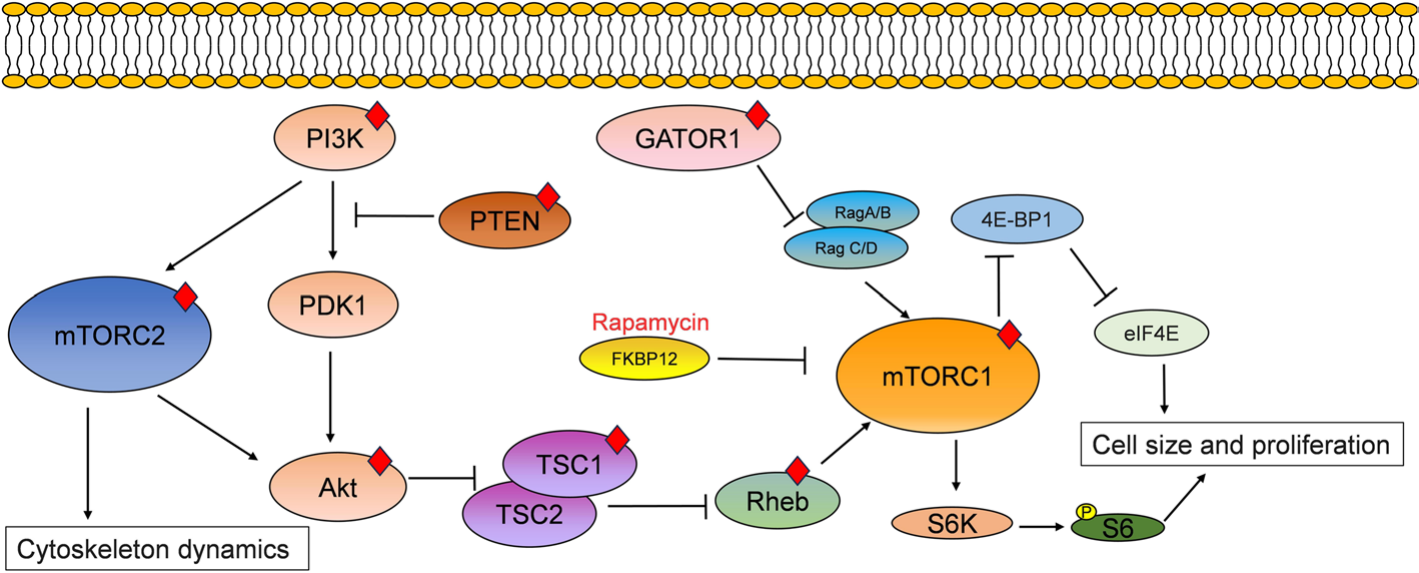
The mTOR pathway and its regulators. mTOR is a protein kinase present in the cell in two complexes: mTORC1 and mTORC2. mTORC1 is related to cell size and proliferation, whereas mTORC2 influences cytoskeleton dynamics. Activation of the PI3K-Akt pathway leads to the inactivation of the TSC1/TSC2 complex, resulting in the indirect activation of mTORC1. PTEN inhibits the PI3K-Akt pathway. Furthermore, PI3K signaling also activates mTORC2. The GATOR1 complex, composed of DEP domain-containing protein 5 (DEPDC5), nitrogen permease regulator-like 2 (Nprl2), and nitrogen permease regulator-like 3 (Nprl3), inhibits mTORC1 signaling by repressing Ras-related GTP-binding protein A/B (RagA/B). Rapamycin binds to FK506-binding protein 12 (FKBP12) and inhibits mTORC1. Red diamonds indicate the proteins whose corresponding genes are mutated in FCD II

Studies have reported the existence of a “mutation gradient”, indicating that the mutational burden is higher at the bottom of the sulcus compared to the gyral crown [83]. Over the past decades, sequencing studies have identified both single-hit somatic mosaicism and two-hit germline plus somatic mutations in genes regulating the mammalian target of the mTOR pathway in FCD II (Fig. 3) [55, 84]. These genes include *PIK3CA* [85], *PTEN* [86], *TSC1/TSC2* [87], GATOR1-encoding complex (*DEPDC5*, *NPRL2*, *NPRL3*) [88–90], *RHEB* [91] and *MTOR* itself [92]. Somatic gain-of-function variants in *MTOR* and its activators, as well as germline, somatic and two-hit loss-of-function variants in its repressors, can culminate in the hyperactivation of the mTOR-signaling cascade [40]. Notably, the genotype-phenotype correlation is increasingly being investigated. Patients with brain somatic *MTOR* variants typically exhibit larger lesions on MRI, including involvement of the white matter. In contrast, patients with GATOR1 complex variants often display subtler MRI visibility and relatively poorer postoperative outcomes. A subset of these patients with GATOR1 complex variants also show a unique and predominantly vacuolizing phenotype, characterized by p62 immunofluorescent aggregates within autophagosomes [93].

DNA sequencing of cerebral samples obtained from subjects with hemimegalencephaly and FCD II has demonstrated that pathogenic somatic variants may arise in both neuronal and non-neuronal lineage. But in minor FCD II instances, these aberrations manifest exclusively within the neuronal lineage [87]. Subsequent to laser capture microdissection of aberrant tissue formations with confirmed gene mutations in the mTOR pathway, a pronounced enrichment of variant allele frequency, reaching up to 45%, was observed in both pools of DNs and BCs compared to morphologically normal neurons (down to 1.4%), and glial cells (down to 1.4%), or bulk tissue [40]. Collectively, these findings indicate that DNs and BCs serve as the primary vectors for mTOR pathway hyperactivating variants in FCD II. Some identified gene mutations in the mTOR pathway recurrently found in patients were engineered into the developing mouse cortex utilizing in utero electroporation techniques, and successfully generated the mouse model recapitulating all neuropathological features of FCD II patients, such as migration defect, DNs, and spontaneous seizures [94–96]. Additionally, it has been substantiated that the inhibition of hyperactivated mTOR kinase can mitigate epilepsy and impede the development of cytomegalic neurons [94]. These genetic discoveries confirmed that de novo gene mutations in mTOR pathway are associated with focal cortical malformation [84].

Currently pathogenic variants in mTOR pathway-related genes can only be detected in 60% of FCD II cases, but the panel-negative FCD II cases also display pS6-positive DNs and BCs [84]. Besides, there is no significant difference in pS6 expression between mutated and non-mutated FCD IIb cases, nor are there significant differences in the clinical manifestations among these patients [97].

The primary reason is the difficulty in detection. These somatic mutations are typically confined to a subset of cells within the brain, making detection through blood or cerebrospinal fluid (CSF) samples less effective; therefore, access to brain tissue is required [98]. Since the mutations arise later in development, they are present in only a smaller fraction of cells. In fact, in 79% of reported cases of mutated FCD II, the brain mosaicism rate is less than 5%, which makes conventional sequencing methods insufficient for detecting low-level mosaic mutations [40]. To address this limitation, numerous novel techniques have now emerged, including fluorescence-activated cell sorting, droplet digital PCR, and novel depth electrode harvesting technique [99–101]. The application of these advanced technologies aids in the detection of ultra-low levels of cellular mutations, holding promise for improving the genetic diagnostic rate within FCD II cohorts.

Besides, there may be undiscovered gene mutations related to FCD II. By integrating whole exome sequencing with amplicon sequencing, researchers identified some new variants not previously linked to FCD. For example, a somatic missense variant in *MED12*, a gene associated with neurodevelopmental syndrome and an upstream regulator of the mTOR pathway, was identified in a patient with FCD IIa [102]. Variants in *IRS1*, *RAB6B*, *ZNF337*, *RALA*, and *HTR6* were also detected. Subsequent in vitro functional studies demonstrated that the *IRS1* variant led to hyperactivation of mTOR [103] and the mutant *IRS1* variant (*IRS-1* c.1791dupG) elicited hyperactivity in phosphorylated extracellular signal-regulated kinase (p-ERK) and augmented cellular volume through the mitogen-activated protein kinase (MAPK) signaling pathway [104]. These findings suggest that a wide range of undiscovered genetic mutations may contribute to the development of FCD II, highlighting the need for further exploration.

### Epigenetic alterations

Epigenetic mechanisms, which represent heritable modifications in gene expression that do not result from an alteration in the DNA sequence, may modulate gene expression and be accountable for some mutation-negative patients [105]. DNA methylation and microRNAs are two epigenetic mechanisms that have been identified in FCD II.

In the mammalian genome, DNA methylation is an epigenetic mechanism achieved by the enzymatic addition of a methyl group (-CH3) from S-adenosyl methionine to the fifth carbon position of cytosine in a CpG dinucleotide [106]. The processes of DNA methylation and hydroxy-methylation impact the proliferation and differentiation of neural stem cells, thereby playing a critical role in the normal development and function of the brain [107]. A comprehensive analysis of DNA methylation and RNA sequencing in FCD II tissues has elucidated the relationship between promoter methylation and gene expression in a subset of genes implicated in receptor tyrosine kinase (RTK) and mTOR signaling, synaptic transmission, and neuronal development and cell-cell interactions. Furthermore, it has been observed that the expression levels of DNA methyltransferase 3α (DNMT3α) are significantly upregulated in patients with FCD II, which may epigenetically regulate the expression of genes involved in cortical development and neuronal plasticity [108]. However, Kobow et al. discovered no significant enrichment of differential DNA methylation or gene expression in mTOR pathway-related genes across different subtypes of FCD, whereas DNs were present in FCD II only [109]. The generation of FCD II may involve complex DNA methylation processes.

MicroRNAs (miRNAs) are a class of single-stranded, noncoding RNAs that function as endogenous posttranscriptional regulators of gene expression by targeting selected protein-coding mRNAs [110]. Recent studies have uncovered a strong association between miRNAs and FCD II. Lee et al. performed miRNA microarray analysis on surgical specimens from cortical dysplasia patients and normal control children who underwent surgical treatment, identifying differentially expressed miRNAs. When the putative target mRNAs were annotated and analyzed by cell signaling pathway, numerous upstream and downstream genes within the mTOR signaling cascade were identified as prospective target entities [111]. Beyond the miRNAs connected to target genes within the mTOR pathway, those correlated with the LIS1 and Hippo pathways have also been pinpointed [111, 112]. LIS1 is crucial for neuronal migration [113], and the Hippo signaling pathway is a regulator of cell proliferation, apoptosis, and differentiation [114]. As a member of the basic helix-loop-helix transcription factor family, NEUROG2 plays a crucial role in determining cell fate and promoting neuronal differentiation [115], exhibiting pronounced nuclear expression in BCs and DNs, related to miRNA regulation. Disruption of the interaction between miR-34a and NEUROG2 precipitated an upregulation in the expression of NEUROG2, thereby compromising the inhibition of neurogenesis and culminating in aberrant neuronal migration and differentiation, ultimately resulting in the formation of FCD II [105, 116]. In summary, epigenetic alterations will also be a key focus in researching the causes of FCD II.

### Cortical developmental protein disturbances

The deregulation in the expression levels and alterations in the cellular distribution of numerous proteins linked to cortical development have been observed in FCD II specimens [54].

Extensive studies have documented elevated levels of numerous growth factors in DNs, encompassing members of the vascular endothelial growth factor (VEGF) family and their receptors [117, 118], the fibroblast growth factor (FGF) family and their receptors [119, 120], along with epidermal growth factor receptor (EGFR) and high-affinity neurotrophin receptors [121, 122]. As pivotal components of the VEGF signaling pathway, the aberrant expression of VEGFA, VEGFB, VEGFC, and their receptors in DNs may potentially impact neuronal differentiation and aberrant NMDA receptor-mediated currents, thereby contributing to the pathogenesis of cortical lesions in patients. Furthermore, the aberrant expression of the FGF family and their receptors not only reflects the putative perturbations of developmental events during the early fetal period that underlie the pathogenesis of these dysplastic lesions [123], but also may lead to the activation of the mTOR pathway [124] and the aberrant expression of bone morphogenic protein-4 (BMP-4) [125].

Bone morphogenetic proteins (BMPs), a significant subfamily within the transforming growth factor-β (TGF-β) superfamily, are multifunctional ligands crucial for regulating intricate cellular processes, such as cell proliferation, differentiation, chemotactic migration, and apoptosis [126]. In comparison to the control group, the FCD II samples showed a reduction in the overall immunoreactivity of BMP-4 staining in their dysplastic cortices. However, moderate to strong BMP-4 immunoreactivity was still observed in malformed neurons, particularly DNs and BCs [127].

Doublecortin-like kinase (DCLK) and its splice variant DCL, play pivotal roles in the orchestration of neurogenesis, neuronal migration, and axonal outgrowth [128]. As a microtubule-associated protein, DCL is prominently expressed in neuronal progenitor cells within the fetal brain and has been demonstrated to be crucial for the microtubule-guided transport of signaling proteins to the nucleus [129]. The pronounced postnatal manifestation of DCL in DNs and BCs underscores the potential relevance to the pathogenesis of FCD II [130].

Nogo-A, a member of the reticulon protein family, is expressed in oligodendrocytes of the adult central nervous system and in developing neurons, serving as a potent inhibitor of neurite outgrowth [131]. In comparison to autopsy control samples, the messenger RNA and protein levels of the Nogo-A receptor (NgR) and its downstream targets, leucine-rich repeat and immunoglobulin-like domain-containing protein 1 (LINGO-1), tumor necrosis factor receptor superfamily member 19 (TROY), and Ras homolog family member A (RhoA), are upregulated in the cortices of FCD II patients. Immunohistochemical analyses have revealed robust expression of Nogo-A and NgR in aberrantly shaped cells, with a particular emphasis on DNs [132]. Additionally, the human leukocyte immunoglobulin-like receptor B2 (LILRB2) signaling pathway, regulated by Nogo-A, is highly expressed in DNs and BCs [133], thereby inhibiting neurite regeneration and prolongation [134]. Collectively, these findings suggest that the Nogo-A system may play a pivotal role in the development and/or seizure activity associated with cortical lesions in FCD II.

Within BCs, the heightened cytoplasmic presence of the dishevelled (Dvl-1) protein was concomitant with the absence of Notch-1, augmented levels of adenomatous polyposis coli (APC), modified cytoplasmic β-catenin, and reduced nuclear β-catenin expression [135]. These findings align with the concept that Wnt/Notch signaling pathways play a pivotal role in neurogenesis, neuroglial cell fate determination, neuronal migration and neural tube development. This suggests disruptions in Wnt/Notch signaling may contribute to the neuropathology of FCD II [54].

Cortical tissue from patients with FCDII exhibited elevated levels of filamin A (FLNA), a consequence of increased transcriptional activity downstream of hyperactive MEK/MAPK signaling, independent of mTOR pathways [136]. Furthermore, reducing FLNA expression alleviated misplacement and dysmorphogenesis [137]. Therefore, targeting FLNA may offer a therapeutic approach that circumvents mTOR dependency.

The aberrant expression of these proteins involved in cortical development regulation may disrupt neuronal-glial proliferation and migration, resulting in the formation of abnormal cortex. However, the causes and mechanisms underlying the abnormal expression of these proteins remain to be explored. Therefore, gaining a more profound insight into these aberrant proteins could offer novel perspectives for therapeutic intervention.

### Inflammatory processes

DNs are recognized as hallmark features of FCD II; however, similar cells are also occasionally observed in certain acquired epilepsy-associated pathologies, such as Rasmussen’s encephalitis (RE)—a rare, chronic inflammatory disorder characterized by intractable seizures during childhood [138]. In RE specimens, the pyroptosis pathway in neurons was mediated by nucleotide-binding oligomerization domain-like receptor family pyrin domain-containing 1 (NLRP1) and NLRP3 inflammasomes, involving activated caspase-1 and increased gasdermin D (GSDMD) expression, which can promote the release of the inflammatory cytokine such as interleukin-1β (IL-1β) [139]. In FCD II cases, phosphorylated phosphoinositide-dependent kinase-1 (pPDK1) and phosphorylated Akt (pAkt), markers of mTOR pathway activation, were expressed only in a subset of pS6-positive DNs, but not in the abnormal cells of RE specimens. The pS6 and IL-1β were co-expressed in DNs that did not exhibit upstream activation of the mTOR pathway [140]. Recent research indicates an upregulation of the expression of the purinergic ionotropic P2X7 receptor (P2X7R) [141] and nuclear factor-κB (NF-κB) p65 accumulates in the nuclei of selected subpopulations of DNs and BCs in FCD IIb and TSC [142]. IL-1β, a downstream factor of the P2X7R signaling pathway, and the canonical NF-κB pathway could lead to the excessive production of inflammatory mediators [143]. Additionally, high-level expression of the inflammatory cytokine interleukin 2 (IL-2) and its receptors were also localized in the malformed cells in FCD II [144]. Furthermore, FCD II tissue shows upregulation of the high-mobility group box 1-Toll-like receptor 4 (HMGB1-TLR4) pathway, resulting in increased downstream pro-inflammatory cytokines, and downregulation of formyl peptide receptor 2, which impairs the neuroinflammation resolution [145, 146]. Activation of the classical complement pathway and microglial reactivity have also been found to be associated with abnormalities in FCD II tissue [147]. Notably, BCs may be pivotal drivers of inflammation, which could partially elucidate why the alterations in FCD IIa are less severe [8].

**Fig. 4:**
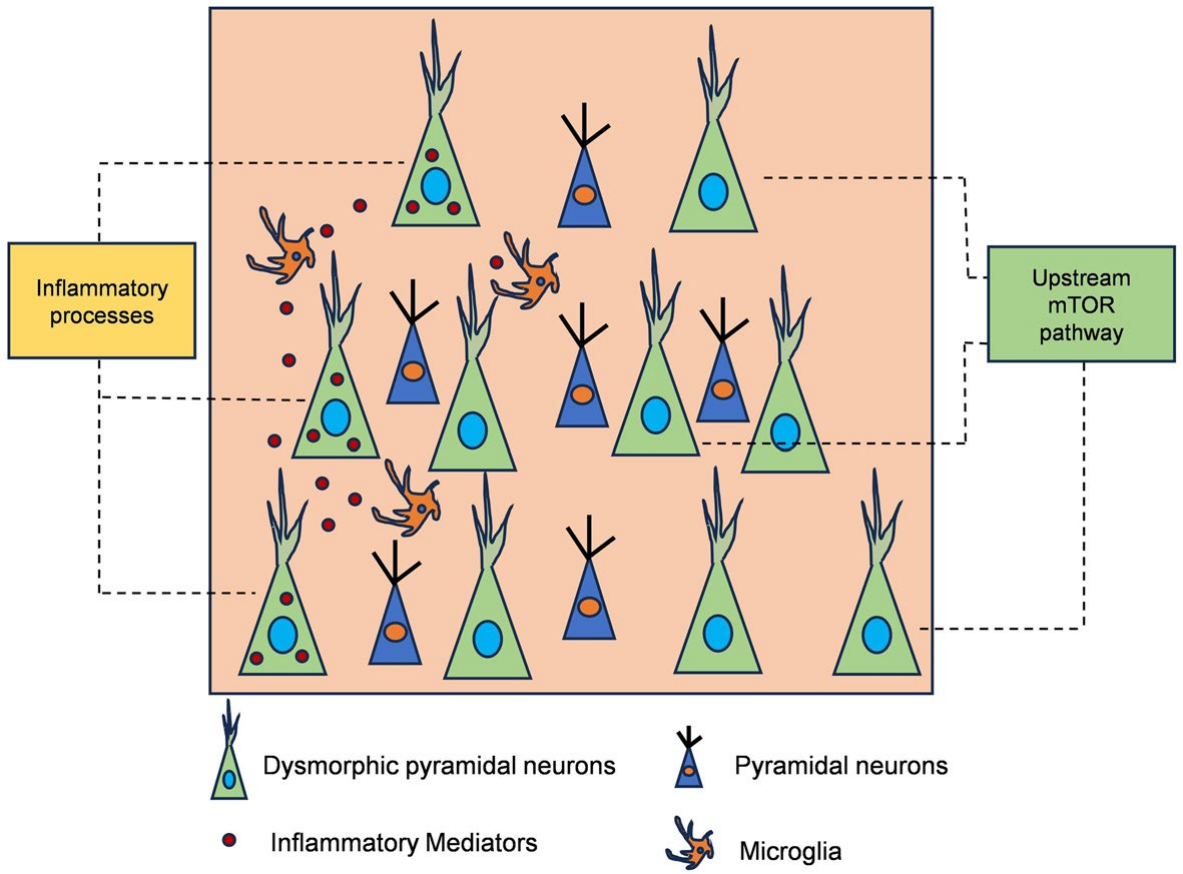
Two hypothesized pathogenetic mechanisms underlying the presence of pS6-positive DNs in FCD II include: (1) activation of the upstream mTOR pathway, and (2) involvement of inflammatory processes

Inflammatory mediators produced during inflammatory processes, such as pro-inflammatory cytokines, can phosphorylate TSC1, leading to the inhibition of the TSC1/2 complex and subsequently activation of mTORC1 [148]. Due to the inflammatory processes may cause the emergence of pS6-positive DNs bypassing the upstream mTOR pathway, the interplay between inflammatory environment and neuroplasticity could be responsible for the etiology of FCD II (Fig. 4) [140].

### Extrinsic damages

A clinical study has found that 32% of patients with cortical malformations have identifiable antenatal risk factors [149]. Thus, a hypothesis posits that extrinsic damages to the developing brain may lead to a focal, field-like defect in cortical maturation [68].

Experimental evidence suggests that animals exposed to physical or chemical factors during early developmental stages exhibit a convergence of pathological phenotypes observed in FCD. For instance, in the rat model of freeze lesioning performed at embryonic day 18, the laminar structure was found to be completely disrupted, with neurons of varying sizes exhibiting disorganized orientation [150]. Addtionally utero X-ray radiated rats showed some neurons losing cortical polarity in morphology at 30 days after birth [118]. Besides, prenatal administration of a double methylazoxymethanol intraperitoneal injection to pregnant rats leads to cellular dysmorphology and laminar disorganization [151]. Similarly, in utero exposure to 1-3-bis-chloroethyl-nitrosurea results in cortical heterotopias and laminar disorganization, accompanied by cytomegalic neurons [152]. This indicates that prenatal exposure to physical or chemical factors can also disrupt the development of the fetal cortex, leading to the formation of cellular abnormalities. However, these cytomegalic neurons did not exhibit clear abnormal expression of pS6, suggesting that the connection between extrinsic damages and FCD II needs additional research [153].

## Conclusions

In this review, we summarize the neuropathological manifestations and pathogenetic mechanisms of FCD II. The neuropathological characteristics, including morphological alterations and specific immunophenotypes, suggest that DNs and BCs exhibit a diverse spectrum of mixed differentiation states. Furthermore, the formation of FCD II is associated with abnormal neuronal proliferation and migration during cortical development. Previously, mutations in the mTOR signaling pathway were considered the primary cause of FCD II. However, mutations in other pathway genes, epigenetic changes, cortical developmental protein disturbances, inflammatory processes, and extrinsic damages have also been linked to the formation of FCD II abnormal tissue. These factors may not be isolated but interconnected. Here, we summarize current research on the pathogenetic mechanisms of FCD II, providing directions for future treatments. Going forward, further research on FCD II abnormal tissue at the single-cell level is needed. A combination of various methods including neuropathology, molecular genetics, cell biology, and computational biology can be utilized to delve deeper into FCD II.

## Data Availability

Availability of data and materials is not applicable in this study.

## Abbreviations

BCs: Balloon cells
BMP: Bone morphogenetic protein
DCLK: Doublecortin-like kinase
DNs: Dysmorphic neurons
FCD: Focal cortical dysplasia
FCD II: Focal cortical dysplasia type II
ILAE: International League Against Epilepsy
MCD: Malformations of cortical development
MTOR: Mechanistic target of rapamycin
pS6: Phospho-S6
RE: Rasmussen’s encephalitis
RGCs: Radial glial cells
SVZ: Subventricular zone
TSC: Tuberous sclerosis complex
VZ: Ventricular zone
XAR: X-androgen receptor
